# Coherence enhancement via a diamond-graphene hybrid for nanoscale quantum sensing

**DOI:** 10.1093/nsr/nwaf076

**Published:** 2025-03-08

**Authors:** Yucheng Hao, Zhiping Yang, Zeyu Li, Xi Kong, Wenna Tang, Tianyu Xie, Shaoyi Xu, Xiangyu Ye, Pei Yu, Pengfei Wang, Ya Wang, Zhenhua Qiao, Libo Gao, Jian-Hua Jiang, Fazhan Shi, Jiangfeng Du

**Affiliations:** CAS Key Laboratory of Microscale Magnetic Resonance and School of Physical Sciences, University of Science and Technology of China, Hefei 230026, China; CAS Center for Excellence in Quantum Information and Quantum Physics, University of Science and Technology of China, Hefei 230026, China; CAS Key Laboratory of Microscale Magnetic Resonance and School of Physical Sciences, University of Science and Technology of China, Hefei 230026, China; CAS Center for Excellence in Quantum Information and Quantum Physics, University of Science and Technology of China, Hefei 230026, China; School of Biomedical Engineering and Suzhou Institute for Advanced Research, University of Science and Technology of China, Suzhou 215123, China; CAS Key Laboratory of Strongly-Coupled Quantum Matter Physics and Department of Physics, University of Science and Technology of China, Hefei 230026, China; The International Center for Quantum Design of Functional Materials, University of Science and Technology of China, Hefei 230026, China; National Laboratory of Solid State Microstructures and Department of Physics, Nanjing University, Nanjing 210093, China; National Laboratory of Solid State Microstructures and Department of Physics, Nanjing University, Nanjing 210093, China; CAS Key Laboratory of Microscale Magnetic Resonance and School of Physical Sciences, University of Science and Technology of China, Hefei 230026, China; CAS Center for Excellence in Quantum Information and Quantum Physics, University of Science and Technology of China, Hefei 230026, China; CAS Key Laboratory of Microscale Magnetic Resonance and School of Physical Sciences, University of Science and Technology of China, Hefei 230026, China; CAS Center for Excellence in Quantum Information and Quantum Physics, University of Science and Technology of China, Hefei 230026, China; CAS Key Laboratory of Microscale Magnetic Resonance and School of Physical Sciences, University of Science and Technology of China, Hefei 230026, China; CAS Center for Excellence in Quantum Information and Quantum Physics, University of Science and Technology of China, Hefei 230026, China; CAS Key Laboratory of Microscale Magnetic Resonance and School of Physical Sciences, University of Science and Technology of China, Hefei 230026, China; CAS Center for Excellence in Quantum Information and Quantum Physics, University of Science and Technology of China, Hefei 230026, China; CAS Key Laboratory of Microscale Magnetic Resonance and School of Physical Sciences, University of Science and Technology of China, Hefei 230026, China; CAS Center for Excellence in Quantum Information and Quantum Physics, University of Science and Technology of China, Hefei 230026, China; Hefei National Laboratory, University of Science and Technology of China, Hefei 230088, China; CAS Key Laboratory of Microscale Magnetic Resonance and School of Physical Sciences, University of Science and Technology of China, Hefei 230026, China; CAS Center for Excellence in Quantum Information and Quantum Physics, University of Science and Technology of China, Hefei 230026, China; Hefei National Laboratory, University of Science and Technology of China, Hefei 230088, China; CAS Key Laboratory of Strongly-Coupled Quantum Matter Physics and Department of Physics, University of Science and Technology of China, Hefei 230026, China; The International Center for Quantum Design of Functional Materials, University of Science and Technology of China, Hefei 230026, China; National Laboratory of Solid State Microstructures and Department of Physics, Nanjing University, Nanjing 210093, China; School of Biomedical Engineering and Suzhou Institute for Advanced Research, University of Science and Technology of China, Suzhou 215123, China; School of Physical Science and Technology & Collaborative Innovation Center of Suzhou Nano Science and Technology, Soochow University, Suzhou 215006, China; CAS Key Laboratory of Microscale Magnetic Resonance and School of Physical Sciences, University of Science and Technology of China, Hefei 230026, China; School of Biomedical Engineering and Suzhou Institute for Advanced Research, University of Science and Technology of China, Suzhou 215123, China; Hefei National Laboratory, University of Science and Technology of China, Hefei 230088, China; Anhui Province Key Laboratory of Scientific Instrument Development and Application, University of Science and Technology of China, Hefei 230026, China; CAS Key Laboratory of Microscale Magnetic Resonance and School of Physical Sciences, University of Science and Technology of China, Hefei 230026, China; Hefei National Laboratory, University of Science and Technology of China, Hefei 230088, China; Anhui Province Key Laboratory of Scientific Instrument Development and Application, University of Science and Technology of China, Hefei 230026, China; Institute of Quantum Sensing and School of Physics, Zhejiang University, Hangzhou 310027, China

**Keywords:** quantum coherence, nanoscale, graphene, hybridization, NV center

## Abstract

Quantum coherence serves as a crucial quantum resource for achieving high-sensitivity quantum sensing. Because of its long coherence time at room temperature, the nitrogen-vacancy (NV) center has emerged as a quantum sensor in various fields in recent years. While nanoscale quantum sensing at room temperature has been demonstrated for NV centers, noise on the diamond surface severely limits its further development at a higher sensitivity. Here, we utilize the hybridization between graphene and diamond surfaces to directly deplete surface unpaired electron spins, thereby achieving roughly two-fold enhancement in coherence. Through the combination of electron spin resonance spectra and first-principle calculations, we explain that this phenomenon arises from a significant reduction in electron spin density on the diamond surface due to interface electron orbital hybridization. Our research presents a new approach for solid-state quantum sensors to reach the desired sensitivity level and offers a new pathway for future studies on material interfaces.

## Teaser

This study establishes a significant enhancement in the spin coherence properties of shallow nitrogen-vacancy centers through graphene-diamond heterostructure integration, proposing a novel methodology to improve sensitivity in solid-state quantum sensing platforms.

## INTRODUCTION

Quantum sensors [1] in solid-state systems have demonstrated their ability to achieve high-sensitivity measurements utilizing quantum resources in a wide range of fields and conditions. In particular, the nitrogen-vacancy (NV) center in diamond [2,3], with its atomic size and long spin coherence even at room temperature, has been highly regarded and extensively explored. Its coupling with various physical quantities enables multifunctional applications in stress [4], magnetic field [5,6], electric field [7] and temperature [8] metrology. In most cases, sensitivity strongly depends on the coherence time of the sensor [9]. Thus, long coherence time is crucial for NV center–based quantum sensing. In many applications of nanoscale signal detection, such as nanoscale nuclear magnetic resonance [10,11] and single-molecule magnetic resonance [12–14], to enhance coupling between the NV center and the sample and achieve higher spatial resolution, NV centers should be created as shallow as possible. However, dangling bonds and other paramagnetic defects at the diamond surface introduce magnetic noise near the NV center, predominantly causing decoherence. Early efforts have been made to control NV centers through microwave decoupling techniques [15,16] or drive surface spins [17] to mitigate surface-induced decoherence, but limitations arise due to rapid heat accumulation from increased microwave duty cycles, restricting practical use. There are also numerous studies that focus on influencing coherence through surface modification [18–21], yet these methods are often inefficient or may affect the detection of outside samples. Recently, Zheng *et al.* [22] achieved coherent enhancement of shallow NV centers by locally controlling the diamond surface spins. While this innovative technique is effective, it comes with various limitations such as requiring complex operations via atomic force microscopy, instability and difficulty in simultaneously improving the coherence of a large batch of NV centers.

In this work, we introduce a novel avenue to enhance coherence via graphene-diamond interactions on their interface, revealing a substantial average enhancement of about 2 times for shallow NV centers (depth $< 20$ nm), with some instances showing improvements of over 2.5 times. By performing double electron-electron resonance (DEER) detection, the density and relaxation time of the surface spin bath are calibrated quantitatively and we attribute the coherence enhancement to the reduction of the surface spin density by graphene-diamond interactions.

## RESULTS

**Figure 1.**
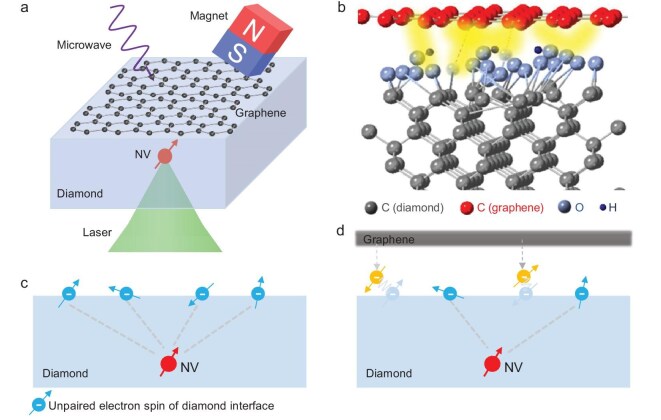
Depletion of unpaired electron spins on the diamond surface with graphene deposition. (a) Schematic illustration of the measurement. A single layer of graphene is transferred to the diamond surface. The underlying NV center is modified and can be used to probe the surface electrons. (b) The unpaired electron spins on the diamond surface interact with the electrons of graphene when graphene is deposited on diamond. (c, d) Schematics showing the interaction of interface electrons before (c) and after (d) graphene deposition. The unpaired electron spins on the diamond contribute to the main decoherence of the NV center.

Unlike three-dimensional (3D) materials including diamond, two-dimensional materials, such as graphene, exhibit an atomically smooth surface with minimal dangling bonds, as exemplified by its $sp^2$ hybridization in graphene [23,24]. This unique structure, while advantageous for electrical properties, can be influenced by substrate interfaces and contacts. When graphene was tightly adhered to the diamond, their interaction depleted unpaired electron spins on the diamond surface (Fig. 1d), contributing to a notable extension of $T_2$. By the comprehensive and quantitative analysis with fitting, we note that the local surface spin density over the NV center decreased significantly and the surface noise was suppressed. The simplicity and practicality of this method stem from the well-established graphene transfer process [25]. CVD-grown graphene is large enough to cover the diamond surface entirely [26], facilitating batch coherence enhancement for NV centers. The structure’s stability under ambient conditions [27] and its atomic thinness ensure the method’s durability and suitability for sensing applications.

A schematic of the experimental principle is illustrated in Fig. 1a. The diamond is a 50-$\mu$m $\langle 100\rangle$-oriented film, including a 10-$\mu\mathrm{m}\, ^{12}\mathrm{C}$-enriched layer. NV centers located at a few to 30 nm below the diamond surface are created by ${ }^{15}\mathrm{N}^{+}$ low-dosage ion implantation and subsequent annealing. Monolayer graphene was directly deposited on the diamond by employing a wet transfer method after acid cleaning and sample-free measurements (see Section A.1 within the online supplementary material). The presence of graphene can be verified by monitoring the decline in NV fluorescence intensity, which is facilitated by Förster resonance energy transfer (FRET) [28]. The comparison of surface electron signals detected by the NV center before and after graphene deposition allows for the assessment of the surface spin density alteration resulting from interface interactions (Fig. 1b).

**Figure 2.**
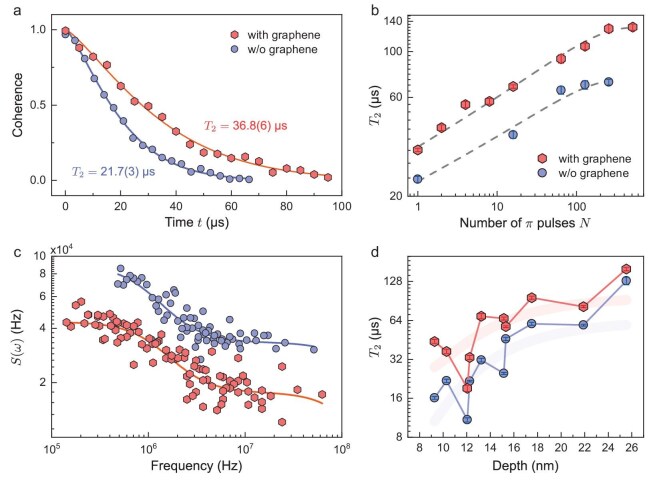
The coherence measurement and noise spectra for NV1 with and without graphene deposited. (a) Hahn-echo measurement demonstrates an enhancement of the coherence time from 21.7(3) to 36.8(6) $\mu\mathrm{s}$. All of the experiments are performed at 382 G, and the data are fitted using $e^{-(t/T_2)^p}$, with *p* from 1.29(4) to 1.33(3). (b) Coherence time as a function of the number of $\pi$ pulses of the dynamical decoupling sequence with and without graphene. (c) Noise spectra derived from decoherence without and with graphene. The solid lines are fitted with a double Lorentzian curve. (d) The $T_2$ enhancement of NV centers at different depths after graphene deposition. The *x* axis is the depth calibrated with the NMR experiment [29] (see Section A.2 within the online supplementary material). The colored lines are visual guides.

In our experiments, the magnetic noise originating from surface electron spins hindered the coherence process of shallow NV centers. The coherence times of NV centers were measured with the Hahn-echo experiment. Figure 2a shows the measurement of coherence time $T_2$ of NV1 without and with graphene at an external magnetic field $B_0$ = 382 G along the symmetry axis of the NV center. Without graphene, $T_2$ was 21.7(3) $\mu$s when the diamond surface was exposed to air; graphene deposition increased this to 36.8(6) $\mu$s, indicating noise suppression. To delve deeper into the coherence enhancement mechanism, we examined the decoherence behavior and noise spectrum of the NV center. Periodic dynamical decoupling Carr–Purcell–Meiboom–Gill pulse sequences with a varying number of $\pi$ pulses were applied until coherence saturation (see Fig. S5 within the online supplementary material). The data were fitted with $e^{-(t/T_2)^p}$ to determine $T_2$, which revealed a consistently longer coherence time for graphene-deposited NV centers across different sequence orders, as depicted in Fig. 2b. The noise spectra were obtained by spectral decomposition [30], with coherence data deconvolved using the filter function of each pulse sequence (see Section B.2 within the online supplementary material). As shown in Fig. 2c, both spectra exhibited similar trends, but the noise intensity was significantly reduced with graphene, indicating its noise suppression capabilities within the resolved frequency range. A summary of $T_2$ values for 10 NV centers is presented in Fig. 2d. Shallow NV centers (depth $< 20$ nm) experienced an average roughly two-fold coherence enhancement, with a maximum over three-fold enhancement observed.

**Figure 3.**
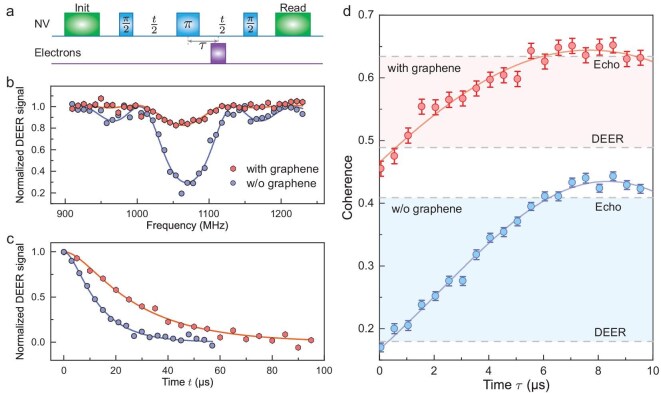
Quantitative measurement of the density and longitudinal relaxation time of the surface electrons by NV1. (a) Schematic of the DEER sequence. (b) Normalized DEER spectra of the diamond sample in air and deposited with graphene. The pulse duration time is fixed, while the frequency of the flip pulse (purple) of electrons is swept from 910 to 1230 MHz. Solid lines are fitted to the analytical solutions under a semiclassical approximation. (c) The DEER decoherence curves are normalized with the noise floor extracted from the noise spectrum to exclude decoherence due to the faster noise, which shows that the coupling strength between the NV center and the surface spin bath decreased from 90.9(50) to 41.0(4) kHz. See the online supplementary material for details. (d) The DEER-echo curves without (blue) and with (red) graphene deposited. The fitting results show that the electron spin density decreased from $2.5(2)\times 10^{-3}$ to $1.1(2)\times 10^{-3}$ nm$^{-2}$, while the relaxation time decreased from 21(3) to 12(3) $\mu\mathrm{s}$. The horizontal dashed lines exhibit agreement with the Hahn-echo and DEER signals.

The observed enhancements in coherence time and reductions in noise levels suggested substantial alterations in the properties of interfacial electron spins. As shown in Fig. 3a, dark spins are measured using the DEER sequence by synchronous control of NV centers and dark spins. DEER spectra shown in Fig. 3b were obtained by sweeping the frequency of the microwave-controlling surface spins. The spectra exhibit a distinct dip at the $g = 2$ resonance frequency of dark electrons without graphene, indicative of the electron spins. Notably, the amplitude of this resonance dip significantly diminished upon graphene coverage, suggesting a suppression of the detected surface electron signal from the interface.

Time-domain DEER signals, as depicted in Fig. 3c, were acquired by varying the free evolution time *t*. The normalized decoherence curves demonstrated a slower decay rate with graphene deposition, indicating a weakening in coupling to surface spins. The curves were fitted with $c(t)=e^{-\gamma _e^{2}B_{\it rms}^{2} \tau _{c}^{2}(e^{-t / \tau _{c}}+t / \tau _{c}-1)}$, where $B_{\it rms}$ is the fluctuating magnetic field induced by electron spins and $\tau _c$ is the electron spin relaxation time. However, extracting precise values for $B_{\it rms}$ and $\tau _c$ using DEER was challenging due to the difficulty in isolating decoherence associated with the surface spin bath (see Section B.3 within the online supplementary material). The fitting results show that the product of these two factors $\gamma _e^{2} B_{\it rms}^2 \tau _c$, called the coupling strength, decreased from 90.9(50) to 41.0(4) kHz.

To further reveal the essence of the interaction mechanism, it is necessary to distinguish the contributions between the density of the interface electrons and their relaxation time. The correlation spectroscopy sequence [31] based on DEER can be used to probe the relaxation time of the surface electron spins, but the sensitivity is not sufficient to acquire an accurate result, especially when the coverage of graphene lowers the density and shortens the relaxation time of electron spins. By fixing the free evolution time and adjusting the delay time $\tau$ of the microwave $\pi$ pulse to target surface spins from the NV $\pi$ pulse, an experimental DEER-echo sequence can be created. This configuration maintains a constant decoherence level, irrespective of the detective spin bath, as $\tau$ varies, effectively isolating spin noise and facilitating the exclusion of other external interference sources. Through fitting the experimental data to the theoretical model (see Equation S16 within the online supplementary material), precise extraction of the spin density and relaxation time can be achieved. As shown in Fig. 3d, the coherence of the DEER-echo measurement is consistent with the DEER experiment at $\tau$ = 0 $\mu\mathrm{s}$ and with the Hahn-echo measurement at $\tau$ = 10 $\mu\mathrm{s}$, in which the sequence degenerates to a DEER or Hahn echo in such conditions. It is worth noting that a distinct peak (coherence exceeds the echo line) appears during the period in each measurement, which shows the susceptibility of the curve shape to the relaxation time $\tau _{c}$. The results indicate a reduction in the electron spin density from $2.5(2) \times 10^{-3}$ to $1.1(2) \times 10^{-3}$ nm$^{-2}$, accompanied by a transition in relaxation time from 21(3) to 12(3) $\mu\mathrm{s}$. Furthermore, a correlation has been noted between the decrease in the electron spin density on alternate NV centers and the extension of $T_2$ (see Fig. S13 within the online supplementary material).

**Figure 4.**
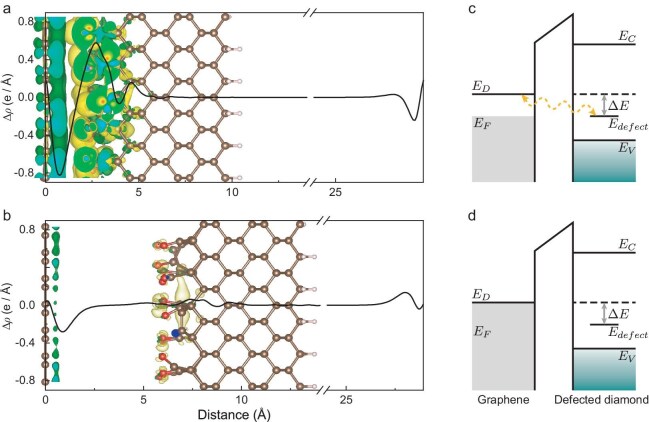
Theoretical analysis of the graphene and diamond interface. (a, b) The visualized charge density difference and planar-average charge density along the *z* direction with a graphene-diamond distance of $2.3\, \mathring{\text{A}}$ in (a) and 6 $\mathring{\text{A}}$ in (b). The balls at $d = 0\, \mathring{\text{A}}$ represent the carbon atoms in graphene, and the ball-stick models that make up the hexagon in the figure are carbon atoms in diamond. The isosurface value is $5\times 10^{-4}$ e/Bohr$^3$. (c) Energy diagram of graphene and diamond with surface defects shows a refined Fermi level and allows charge flows from graphene to diamond. (d) Energy diagram of graphene and diamond when they are far away or not interacting.

To elucidate the effects of graphene on diamond, the charge distribution and orbital projected band structure of the graphene-diamond heterostructure are analyzed by *ab initio* calculations. A graphene monolayer is deposited on the surface of $\langle 100\rangle$-oriented diamond with a graphene-diamond distance of $2.3$ Å after full relaxation, accompanied by residual bonds such as C−, O− and OH− terminals at the diamond surface. The charge redistribution at the interface is illustrated by the charge density difference, which is defined as $\delta \rho = \rho _{\rm dia/gra}-\rho _{\rm dia}-\rho _{\rm gra}$, where $\rho _{\rm dia/gra}$, $\rho _{\rm dia}$ and $\rho _{\rm gra}$ are the charge densities of the diamond/graphene van der Waals (vdW) heterostructure, diamond and graphene, respectively. Figure 4a shows the charge density map of the system, with the black solid line corresponding to the planar-average charge density along the *z* direction.

It is obvious that the charge density redistribution and even overlap are formed in the vdW gap. The charge accumulation regions are close to the side of diamond, demonstrating that electrons can be transferred from graphene to diamond and allow interactions to occur. In contrast, the charge density map with a graphene-diamond distance of 6 Å is shown in Fig. 4b. The charge density map shows that the electrons just accumulate at the surface of graphene without redistribution once the space is large enough. We also calculated the charge transfer of the graphene-diamond interface with different initial distances by using Bader charge analysis [32] (see Fig. S17 within the online supplementary material). The results showed that the transferred electrons monotonically decrease with increasing distance, which could explain the difference in density variations for each NV center. In addition, the projected band structure results suggest that the presence of the diamond substrate has a negligible effect on the energy structure of graphene. For the structures with these defects, the band structure that only comprises *p* orbitals of diamond exhibits typical features of graphene-gapped Dirac cones (see Fig. S18 within the online supplementary material).

Thus, the changes can be explained when we consider the orbital hybridization of electrons between the graphene-diamond interface. When the diamond was not covered with graphene, the electron spins on the diamond surface relaxed due to the surrounding noise. When the graphene was tightly adhered to the diamond, their interactions redefined the position of the Fermi level and the electrons in the graphene tended to the defect energy-level diamond surface and hybridized with the unpaired electrons of the diamond, as shown in Fig. 4c. Because of our room-temperature conditions, the electrons may transition back and forth between graphene and diamond surface bound states, leading to a decrease in the electron spin relaxation time in Fig. 3d. Some highly hybridized graphene-diamond surface electrons paired and formed covalent bonds of spin singlet states, thus reducing the density of electron spins that can be detected by the NV center. In addition, it is well known that the mobility of electrons in graphene is fast with a spin relaxation time estimated to be at the picosecond to nanosecond level [33–35], which is not detectable and thus does not contribute to the decoherence of the NV center in our experiments.

## CONCLUSIONS

In summary, we experimentally observed the enhanced coherence of NV centers in diamond resulting from the graphene-diamond surface interaction. The signal of diamond surface electrons detected decreased following the deposition of graphene onto the diamond surface. Further experimental results and analysis revealed hybridization at the diamond-graphene interface, leading to a decrease in the density of surface electron spins. Furthermore, a more efficient contact between the surfaces, such as annealing under an ultra-high vacuum [36], could potentially enhance this effect. Despite the limiting influence of the FRET effect on achieving higher-sensitivity enhancements, particularly apparent when the NV center depth is under 10 nm, doping has the potential to mitigate this effect and warrants further investigation [26]. More generally, this method may be applicable to other spin-based solid-state quantum systems, such as color centers in SiC [37] and *h*-BN [38]. In addition, our research not only introduces a new approach to coherence enhancement in solid-state systems, but also offers a fresh perspective on investigating atomic level interface interactions. This study exemplifies how a 2D material like graphene can impact a 3D material like diamond through the mediation of a 0D NV defect *in situ*, presenting a unique framework for future explorations of material interfaces [39].

## Supplementary Material

nwaf076_Supplemental_File
