# Supplementary material for: Coherence enhancement via a diamond-graphene hybrid for nanoscale quantum sensing
Source: Natl Sci Rev. 2025 Mar 8;12(5):nwaf076. doi: 10.1093/nsr/nwaf076 (PMC12023861; doi:10.1093/nsr/nwaf076)
Supplement: nwaf076_Supplemental_File [file nwaf076_supplemental_file.pdf]

Supplementary Data for Coherence enhancement via diamond-graphene hybrid for nanoscale quantum sensing

A: Experimental Methods

1. Setup and system

The diamond sample used in our experiments is a 2 mm × 2 mm × 0.05 mm ⟨100⟩-oriented film, consisting of a 10 μm ^{12}C-enriched (99.99% abundance) layer CVD-grown on a naturally abundant diamond substrate. The thin diamond film was implanted using a ^{15} N^+ dosage of 10^9 cm^{-2} with different implantation energies of 7 and 11 keV and following annealed in vacuum. Then the diamond was subjected to an acid treatment (equal parts H2SO4, HNO3 and HClO4 at 180 °C for 3 hours) to clean and oxygen terminate the surface.

The graphene was deposited on Cu-Ni alloy layer by Proton-assisted chemical vapor deposition [1]. We used the standard wet transfer process to transfer the graphene onto the diamond surface [2]. First, an ammonium persulfate solution (2 mol/L) was used to etch the alloy layer, and then the graphene was transferred to pure water to wash away the residual ammonium persulfate. The slide glass used to place the diamond was cleaned in boiling piranha solution (120 °C) to become hydrophilic to drain the water between the graphene and the diamond chip. After transferring the graphene from the water to the diamond surface, the graphene was brought into contact with the diamond surface by annealing in the atmospheric environment with the following sequence:

1. Place at room temperature for 10 minutes;
2. Ramp to 80 °C. Hold for 5 minutes;
3. Ramp to 160 °C.Hold for 10 minutes;
4. Let cool to room temperature.

2. Depth calibration of NV centers

The depth of NV centers was determined by fitting the proton-NMR spectrum to the theoretical model expression with finite T_2^* [3]. The nuclear magnetic signal comes from the proton-rich sample, immersion oil (Fluka Analytical 10976). The Förster resonance energy transfer (FRET) effect between the shallow NV center and graphene is obvious due to their short distance (Fig. S3B).

B: Derivation of the Decoherence Behavior

1. Hahn-echo sequence and Ramsey sequence

For the ^{12}C-purified diamond sample, the decoherence of the NV centers is mainly due to the magnetic noise of the dark electron spins at the diamond surface. In the regime of a low magnetic field and room temperature (relevant to our cases), the noise generated by a uniform spin bath can be described as Lorentzian under the classical approximation [4]. For Lorentzian noise, if the correlation time is τ_c, the autocorrelation function of the noise will be

$$\langle b(t)b(t')\rangle = B_{\mathrm{rms}}^2 e^{-|t-t'|/\tau_c}, \tag{S1}$$

where $b(t)$ is the spin noise detected by the NV center, and B_{rms} is the RMS magnetic field. The phase accumulated of NV in a single detection is

$$\varphi = \int_0^\tau g(t)\gamma_e b(t)dt, \tag{S2}$$

where $g(t)$ is the modulation function and γ_e is the gyromagnetic ratio. The mean of the phase φ is zero, while the variance $\Delta\varphi$ is not. The final result of the experiment is the average of all the detections (more than 10^6 times) to obtain a sufficiently high signal-to-noise ratio (SNR), so the decoherence induced by the magnetic field fluctuation is

$$C = \left\langle e^{i\varphi}\right\rangle = e^{-\left\langle\varphi^2\right\rangle/2} = e^{-\Delta\varphi^2/2}, \tag{S3}$$

$$\Delta\varphi^2 = \frac{\gamma_e^2}{2\pi}\int_{-\infty}^{+\infty} F(\omega)S(\omega)d\omega, \tag{S4}$$

where $F(\omega) = \left|\int_{-\infty}^{+\infty} g(\tau)e^{-i\omega\tau}d\tau\right|^2$ is the filter function of the sequence we used, and $S(\omega) = \int_{-\infty}^{+\infty}\langle b(\tau)b(\tau')\rangle e^{-i\omega\tau}d\tau$ is the noise spectrum. According to Eq. S1, the magnetic noise spectra of the spin bath is $S(\omega) = 2B_{\mathrm{rms}}^2\tau_c/(1+w^2\tau_c^2)$.

For the Hahn-echo sequence, the filter function is $F(\omega) = \frac{16}{\omega^2}\sin^4\left(\frac{w\tau}{4}\right)$, then

$$\begin{aligned}\Delta\varphi^2 &= \gamma_e^2\int_{-\infty}^{+\infty}\frac{16}{\omega^2}\sin^4\left(\frac{\omega\tau}{4}\right)\frac{{B_{\mathrm{rms}}}^2}{\pi}\frac{\tau_c}{1+\omega^2\tau_c^2}d\omega \\ &= 2\gamma_e^2{B_{\mathrm{rms}}}^2\tau_c^2\left(\tau/\tau_c - 3 + 4e^{-\tau/2\tau_c} - e^{-\tau/\tau_c}\right).\end{aligned} \tag{S5}$$

Specially, the result can be approximated as $C(t) \approx e^{-\gamma_e^2 {B_{\rm rms}}^2 t^3/(12\tau_c)}$ when the total evolution time t is much shorter than τ_c, or $C(t) \approx e^{-\gamma_e^2 {B_{\rm rms}}^2 t\tau_c}$ when t is much longer than τ_c. Since decoherence behavior seems to be complex, we use $C(t) \approx exp-(t/T_2)^n$ $(1 \le n \le 3)$ to simplify the model even though the above conditions are not satisfied.

For the Ramsey sequence, the filter function is $F(\omega) = \frac{4}{\omega^2}\sin^2\left(\frac{\omega\tau}{2}\right)$, then

$$\begin{aligned}\Delta\varphi^2 &= \gamma_e^2 \int_{-\infty}^{\infty} \frac{4}{\omega^2}\sin^2\left(\frac{\omega\tau}{4}\right)\frac{{B_{\rm rms}}^2}{\pi}\frac{\tau_c}{1+\omega^2\tau_c^2}d\omega \\ &= 2\gamma_e^2 {B_{\rm rms}}^2 \tau_c^2 \left(e^{-\tau/\tau_c} + \tau/\tau_c - 1\right).\end{aligned} \tag{S6}$$

It is worth noting that the decoherence due to the dark spins in the DEER sequence is the same as in the Ramsey sequence because the manipulations of the dark spins and the NV center are synchronized. Although it is theoretically possible to obtain the exact $B_{\rm rms}$ and τ_c by fitting with the formula, it is not feasible because the decoherence irrelevant to the spin noise is hard to be eliminated completely (Fig. S4). Even so, the DEER method visually shows the coupling strength between the NV center and surface spins as shown in Fig. 3 in the main text.

2. Spectral decomposition and noise spectrum

The filter function of high-order dynamical decoupling sequences can be approximated as [5]:

$$F(\omega) \approx \frac{8t}{\pi}\sum_{k=-\infty}^{+\infty}\frac{\delta(\omega-(2k+1)\omega_0)}{(2k+1)^2}, \tag{S7}$$

where t is the total evolution time and $\omega_0 = \pi N/t$. Combining Eq. S3, S4 and S7, the noise spectral density at frequency ω_0 is given by

$$\frac{8}{\pi^2}\sum_{k=0}^{+\infty}\frac{S((2k+1)\omega_0)}{(2k+1)^2} = \frac{-2lnC(T)}{\gamma_e^2 T}, \tag{S8}$$

In most cases, the lowest order approximation is sufficient, then

$$S(\omega_0) = \frac{-\pi^2 lnC(T)}{4{\gamma_e}^2 T}. \tag{S9}$$

To obtain the wide-range noise spectra, ω_0 should slide on the frequency domain and multiple dynamical decoupling sequences with different orders are executed (Fig. S6).

The noise spectrum were fitted by double Lorentzian function

$$S(\omega) = \sum_{i=1,2}\frac{2\beta_{(i)}^2\tau_{c(i)}}{(1+(\omega\tau_{c(i)})^2)}, \tag{S10}$$

where $\beta_{(i)} = \gamma_e B_{rms(i)}$ is the coupling strength between the noise and NV center, and $\tau_{c(i)}$ is the noise correlation time. Technically, we set $\beta_{(i)}^2\tau_{c(i)}$ as fitting parameters to normalize the DEER signal (See in Section B.3). And the fitting results are $\beta_{(1)}^2\tau_{c(1)} = 28 \pm 4$ kHz,$\tau_{c(1)} = 0.9 \pm 0.2\mu s$,$\beta_{(2)}^2\tau_{c(2)} = 16.7 \pm 0.5$ kHz,$\tau_{c(2)} = 4 \pm 7$ ns and $\beta_{(1)}^2\tau_{c(1)} = 13 \pm 1$ kHz,$\tau_{c(1)} = 0.8 \pm 0.1\mu s$,$\beta_{(2)}^2\tau_{c(2)} = 8.8 \pm 0.3$ kHz,$\tau_{c(2)} = 6 \pm 6$ ns without and with graphene coating, respectively.

3. DEER method

To identify the spins with their g-factor, the DEER sequence shown in Fig. S7 with varying frequency f_s is performed. We also calculated the analytical solution of DEER spectrum to fit the experimental data. Instead of considering the filter function in Section B.1, we calculate $\Delta\varphi^2$ in a more direct way. For the DEER sequence, $\Delta\varphi^2$ takes the form of

$$\Delta\varphi^2 = \left\langle\left(\int_0^{\tau/2}\gamma_e b(t)dt - \int_{\tau/2}^{\tau}\gamma_e b'(t)dt\right)\left(\int_0^{\tau/2}\gamma_e b(t')dt' - \int_{\tau/2}^{\tau}\gamma_e b'(t')dt'\right)\right\rangle, \tag{S11}$$

where τ is the evolution time. When applying a manipulation to the dark spin, we have

$$\langle b(t)b'(t')\rangle = \Delta n \left\langle B_{\rm rms}^2\right\rangle e^{-|t-t'|/\tau_c}, \tag{S12}$$

where Δn is the difference in population. Assuming that the π pulse is perfect when the detuning $\delta\omega$ is zero, Δn takes the form of

$$\Delta n = \frac{\Omega^2}{\Omega^2+\delta\omega^2}\sin^2\left(\frac{\sqrt{\Omega^2+\delta\omega^2}}{2\Omega\pi}\right), \tag{S13}$$

where Ω is the Rabi frequency. We can acquire $\delta\varphi$ by substituting Eq. S12 and S13 into Eq. S11. The final result is

$$\Delta\varphi^2 = 2\gamma_e^2 B_{\rm rms}^2 \tau_c^2(\tau/\tau_c + 2e^{-\tau/2\tau_c} - 2) - 2\Delta n {\gamma_e}^2 B_{\rm rms}^2 \tau_c^2 (e^{-\tau/\tau_c} - 2e^{-\tau/(2\tau_c)} + 1). \tag{S14}$$

According to Eq. S3 and S14, we arrive at the analytical solution of the DEER spectra.

Although the analytical solution of DEER signal is explicit theoretically, the decoherence behavior is more complex in practice due to the noise sources besides the controlled electrons. This noise can't be controlled with the microwave and is direct coupled into the DEER signal, so the whole decoherence behavior is $C(\tau) = \exp(-\Delta\varphi_1^2)\exp(-\Delta\varphi_2^2)$, where $\Delta\varphi_1$ is the phase accumulated due to the controlled electron spin and $\Delta\varphi_2$ is the phase accumulated due to other noise. The aim of normalization is to eliminate decoherence that is unrelated to the controlled surface spin. In a short word, the normalization is carried out by dividing the whole decoherence curve by the decoherence unrelated to the controlled surface spin. Here the principle is briefly shown as below.

At first, we fit the noise spectrum with double Lorentzian function (Eq. S10) as shown in Fig. 2c in the main text and the Section B.2, considering $\beta_i^2\tau_{c(i)}$ and $\tau_{c(i)}$ as fitting parameters. The result shows two distinct components of the noise, the slow one with a longer correlation time($\tau_{c(1)} \sim \mu s$) and the fast one with a shorter correlation time($\tau_{c(2)} \sim ns$). We attributed the fast one to surface-modified phonons or electric field noise, which is uncontrollable and contributes to the baseline noise level in the spectrum. The decoherence behavior due to the fast noise is $C_{(2)}(\tau) \approx \exp(-\beta_{(2)}^2\tau_{c(2)}\tau)$ as $\tau_{c(2)} \ll \tau$, according to Eq. S5.

Secondly, we then divided the total DEER signal by $C_{(2)}$ to exclude the effect of the fast noise. More specifically, the DEER decoherence curve is divided by $\exp[-\tau/(60\mu s)]$ (without graphene) and $\exp[-\tau/(113\mu s)]$ (with graphene) in Fig. 3c, as the $\beta_{(2)}^2\tau_{c(2)}$ without and with graphene are 8.8±0.3kHz and 16.7±0.5kHz respectively.

Indeed, the double Lorentzian model is sample and we can't exclude the uncorrelated noise completely by this method (Fig . S4). Even so, the normalized DEER spectrum show the difference in the coupling strength between the NV and surface spins in a semi-quantitative and intuitional way. To analyze the density and the relaxation time of surface spins more quantitatively, we use the DEER-echo method, which is described in detail in Section B.4.

We also performed the Hahn-echo and DEER experiments with h-BN coating, as shown in Fig. S8. It is obvious that there is no positive effect for h-BN to clear the surface and enhance the coherence time T_2, and the side effect may come from the defect on the single-layer h-BN (dangling band and so on). To verify the theoretical model, numerical simulations were performed, as shown in Fig. S12. The agreement between the results of experiment and numerical simulation shows that the spin bath can be effectively controlled under the current conditions.

4. DEER-echo method

The DEER-echo sequence shown in Fig. S7b is crucial to determine $B_{\rm rms}$ and τ_c. Similar to the derivation above, we can obtain

$$\Delta\varphi^2 = \left\langle \left(\int_0^{2\tau_0}\gamma_e b(t)dt - 2\int_{\tau_0}^{\tau_0+\tau}\gamma_e b(t)dt\right)\left(\int_0^{2\tau_0}\gamma_e b(t')dt' - 2\int_{\tau_0}^{\tau_0+\tau}\gamma_e b(t')dt'\right)\right\rangle. \tag{S15}$$

Assuming that the noise is Lorentzian (Eq.S1), we can obtain $\Delta\varphi$ in a similar way, and the result is

$$\Delta\varphi^2 = 4\tau_c\gamma_e^2 B_{\rm rms}^2\tau_0 - 2\gamma_e^2 B_{\rm rms}^2\tau_c^2[5 - 4e^{-\frac{\tau}{\tau_c}} + 2e^{-\frac{\tau_0}{\tau_c}}(e^{-\frac{\tau}{\tau_c}} - e^{\frac{\tau}{\tau_c}}) - e^{-\frac{2\tau_0}{\tau_c}}]. \tag{S16}$$

Substituting Eq. S16 and Eq. S23 into Eq. S3, we arrive at the analytical solution.

To verify the theoretical model, numerical simulations were performed as shown in Fig. S12. When the data are fitted by Eq. S16, the decoherence caused by the noise that cannot be controlled by microwaves, and can be easily treated as a constant factor before the exponential decay since the total evolution time is a constant. The decoherence irrelevant to the spin noise can be excluded more completely than the DEER method, as shown in

Fig. S4 .

5. Correlation spectroscopy

To verify the reliability of the DEER-echo method, τ_c was also measured with correlation spectroscopy without graphene. The pulse sequence is shown in Fig. S7c.

Unlike the sequence mentioned above, for the correlation spectroscopy, the coherence is

$$C(\tau) = \langle \sin(\varphi_1)\sin(\varphi_2)\rangle \,. \tag{S17}$$

where the φ_1 and $2\varphi_2$ are the accumulated phase in the first and second halves of the sequence, respectively. If φ_1 and φ_2 are small, Eq. S17 becomes

$$C(\tau) \approx \langle \phi_1\phi_2\rangle \,. \tag{S18}$$

Noticing that the sequence is composed of two Hahn-echo sequences and using the results in Section B.3, we can obtain

$$C(\tau) \approx {\gamma_e}^2 B_{\rm rms}^2 \tau_c^2 (1 + e^{4\tau_0/\tau_c} - 2e^{2\tau_0/\tau_c})e^{-\tau/\tau_c}. \tag{S19}$$

An exponential damping curve can be acquired when changing the interval τ between two Hahn-echo sequences. As shown in Fig. S13, the results of correlation spectroscopy is consistent with the DEER-echo method.

Although the correlation spectroscopy shows the correlation time more directly, it has vital limitations, such as the relatively low sensitivity due to the long sequence time. Especially, the contrast suffer greatly from the short relaxation time of the surface spin, which can be seen from the coefficient in front of the exponent in Eq. S19. When the measurement is performed with graphene coated on the diamond, the problem of lower sensitivity is further amplified due to the FRET effect. Taking with NV1, to achieve a sufficiently high SNR, dozens of hours need to be taken for the correlation spectroscopy with graphene deposited, while it takes only a few to ten hours for the DEER-echo sequence, not to mention that the accurate $B_{\rm rms}$ cannot be derived due to the approximation.

C: Additional Approximation and Theoretical Calculation

1. Continuous spin distribution approximation

When obtaining $B_{\rm rms}$ and τ_c by DEER-echo sequence, we can then derive the surface spin density and relaxation time, which are more intuitive physical quantities.

Noticing that the transverse part of the spin moment has a negligible effect on the NV decoherence, the magnetic field generated by the longitudinal moment of a 1/2-spin that we only considered is

$$\vec{b_i} = \frac{\mu_0\hbar\gamma_e}{8\pi|r_i|^3}(\frac{3\vec{r_i}(\vec{r_{0i}}\vec{r_i})}{|r_i|^2} - \vec{r_{0i}}), \tag{S20}$$

where $\vec{r_i}$ is the vector from the position of the ith spin to the point of the space we are interested in, and $\vec{r_{0i}}$ is the unit vector along $\vec{r}$. Furthermore, we were only concerned with the magnetic field parallel to the NV axis which causes NV spin dephasing. We can obtain the magnetic field parallel to the axis according to Eq. S20,

$$b_{\parallel i} = \frac{\mu_0\hbar\gamma_e}{8\pi}\frac{3cos^2\theta - 1}{|r_i|^3}, \tag{S21}$$

where θ is the angle between $\vec{r}$ and the NV axis, as shown in Fig.S15. Suppose the spins are independent of each other and consider a uniformly distributed 2D spin bath where the spin-spin distance is smaller than NV depth d. The sum over all spins can be approximated to an integral, then

$$B_{\rm rms}^2 = \frac{\mu_0\hbar\gamma_e}{8\pi}^2 \sigma \int_{-\infty}^{+\infty}\int_{-\infty}^{+\infty} \frac{(x^2 - y^2 - 2\sqrt{2}xd)^2}{(x^2+y^2+d^2)^5}dxdy, \tag{S22}$$

where σ is the spin density and the final result is

$$B_{\rm rms}^2 = \frac{3\pi}{8d^4}(\frac{\mu_0\hbar\gamma_e}{8\pi})^2\sigma \approx (1.001mT\ nm^3)^2\frac{\sigma}{d^4}. \tag{S23}$$

The spin relaxation time of the spin bath is the correlation time of the spin noise exactly, which can be derived from Eq. S1. For the spin bath, the density matrix can be described as $\frac{1}{2}\hat{I} + n_x\hat{S}_x + n_y\hat{S}_y + n_z\hat{S}_z$, and $b(t) \sim n_z(t)\gamma_e\langle\hat{S}_z\rangle$ (the terms S_x and S_y are suppressed well by rotating-wave approximation), then: $\langle\langle\hat{S}_z(0)\rangle\langle\hat{S}_z(t)\rangle\rangle = \langle\langle\hat{S}_z(0)\rangle^2\rangle exp(-t/\tau_c)$. It's obvious that τ_c can be considered as the longitudinal relaxation time of the electron spin bath.

For NV depths comparable to or below the average spin-spin distance, the approximation of the uniform spin distribution breaks down due to the small number of spins that couple with the NV center. Nevertheless, the fitting results for the correlation time are still reliable, as shown in Fig. S16. Thus, the spin density obtained with Eq. S23 can be considered as the effective spin density

near the NV center.

2. Theoretical calculation

Our first-principles calculations were performed using the projected augmented-wave method as implemented in the VASP package[6], and the generalized gradient approximation exchange correlation[7] potential was used. The kinetic cutoff energy of the plane wave was set to 400 eV. The Brillouin zone was sampled with a Γ-centered 431 grid based on the scheme proposed by Monkhorst and Pack for the calculation of electronic structures[8]. The convergence criterion was set to 10^{-5} eV for energy in both optimization and self-consistent field calculations. Rectangle supercells of both diamond and graphene were used to construct a heterostructure with a small mismatch ($\sim$ 2.3%). Three diamond $\langle 100 \rangle$ surfaces were considered, as C-terminal, O-terminal and COO-terminal. During structural optimization, all atoms were fully relaxed, and the force converged to less than 0.01 eV/Å. A vacuum buffer space over 15 Å was included to prevent interaction between adjacent slabs. Furthermore, the DFT-D3 functional was switched on to correct the dispersion due to van der Waals interactions between graphene and diamond[9].

[1] Yuan G, Lin D, Wang Y *et al.* Proton-assisted growth of ultra-flat graphene films. *Nature* 2020; **577**: 204–208.

[2] Suk JW, Kitt A, Magnuson CW *et al.* Transfer of cvd-grown monolayer graphene onto arbitrary substrates. *ACS nano* 2011; **5**: 6916–6924.

[3] Pham LM, DeVience SJ, Casola F *et al.* Nmr technique for determining the depth of shallow nitrogen-vacancy centers in diamond. *Phys. Rev. B* 2016; **93**: 045425.

[4] Bar-Gill N, Pham LM, Belthangady C *et al.* Suppression of spin-bath dynamics for improved coherence of multi-spin-qubit systems. *Nat. commun.* 2012; **3**: 858.

[5] Xie T, Zhao Z, Kong X *et al.* Beating the standard quantum limit under ambient conditions with solid-state spins. *Sci. Adv.* 2021; **7**: eabg9204.

[6] Kresse G and Furthmüller J. Efficient iterative schemes for ab initio total-energy calculations using a plane-wave basis set. *Phys. Rev. B* 1996; **54**: 11169–11186.

[7] Perdew JP, Burke K and Ernzerhof M. Generalized gradient approximation made simple. *Phys. Rev. Lett.* 1996; **77**: 3865–3868.

[8] Monkhorst HJ and Pack JD. Special points for brillouin-zone integrations. *Phys. Rev. B* 1976; **13**: 5188–5192.

[9] Grimme S, Antony J, Ehrlich S *et al.* A consistent and accurate ab initio parametrization of density functional dispersion correction (DFT-d) for the 94 elements h-pu. *J. Chem. Phys.* 2010; **132**: 154104.

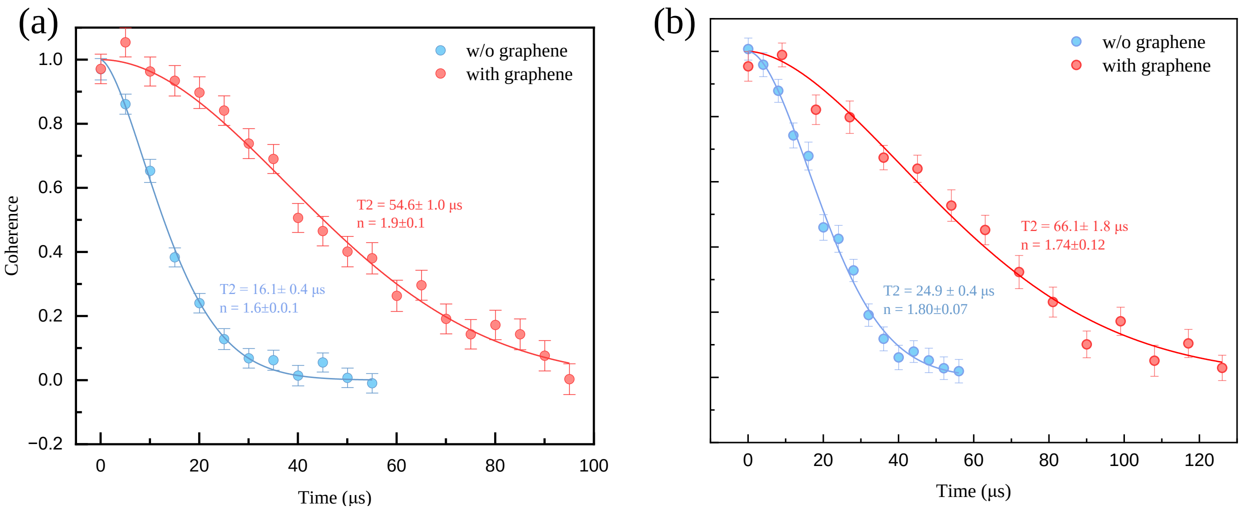

FIG. S1. T_2 **measurement of two NVs.** The T_2 showed a extension of 3.4-folds and 2.7-folds with NV depth of 9.3 ± 0.4 nm(left) and 15.1 ± 0.4 nm(right) respectively.

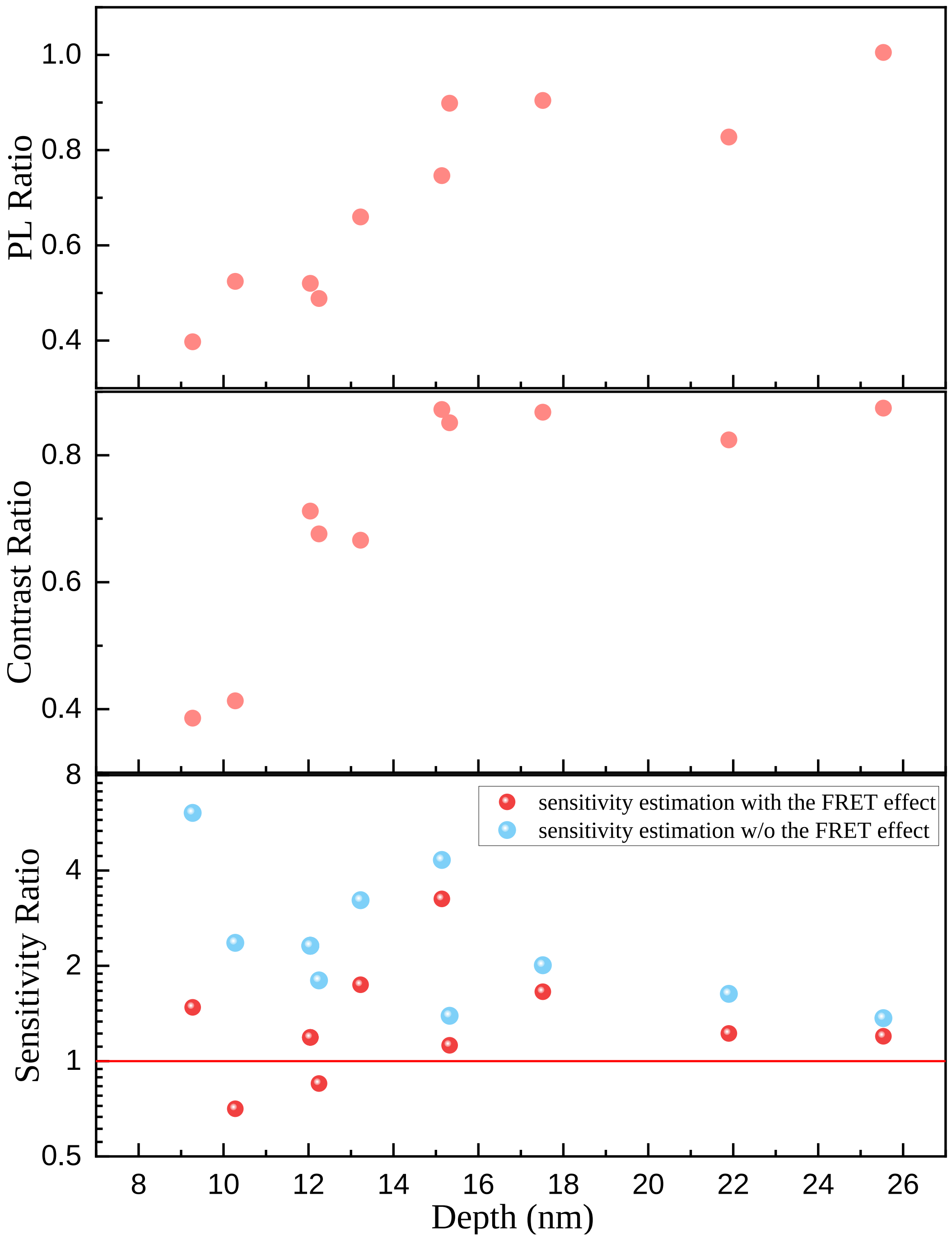

FIG. S2. **Estimation of the sensitivity improvement.** The FRET effect reduces the PL counts(upper) and contrast(middle) of the NV center. The shallower the NV center, the lower the contrast and PL counts. The lower panel shows the estimation of sensitivity ratio after and before graphene treatment. Blue balls show the sensitivity improvement when the FRET effect disappeared. The sensitivity change is comprehensively considered with T_2 enhancement and readout efficiency drop due to the FRET effect according to (sensitivity) $\propto \frac{1}{C\sqrt{n_{avg}}}T_2^{-3/2}$, where C is the contrast and n_{avg} is the average photon number collected. Value greater than one indicates an enhancement. Although the FRET effect hindered us reach the sensitivity considering coherence enhancement only, the sensitivity does have statistical improvements in our measured depth range. Remarkably, we achieved an 3-folds sensitivity enhancement with a NV depth of about 15nm, which corresponds to a 9-folds measurement speed-up.

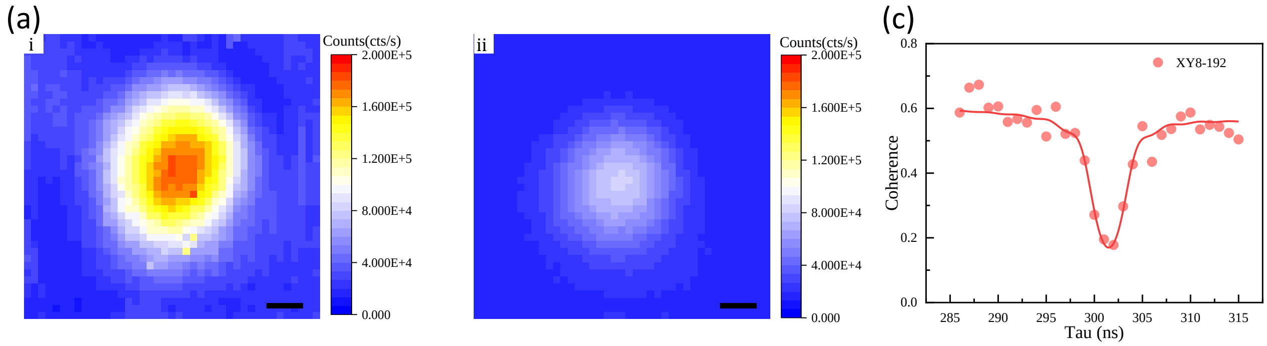

FIG. S3. **An example of the depth calibration of the NV center. (a)** Confocal image of NV1. (i) The NV1 was in the center of the image without graphene, and the spot size showed the optical diffraction limit. (ii) The fluorescence of NV1 was quenched due to the FRET effect with graphene deposited. Both data were acquired under the same laser power. Scale bar: 100 nm. **(b)** The NMR signal of NV1 at 390 Gauss measured with XY8-192. The solid line is a fit to the theoretical model to determine the depth of the NV center.

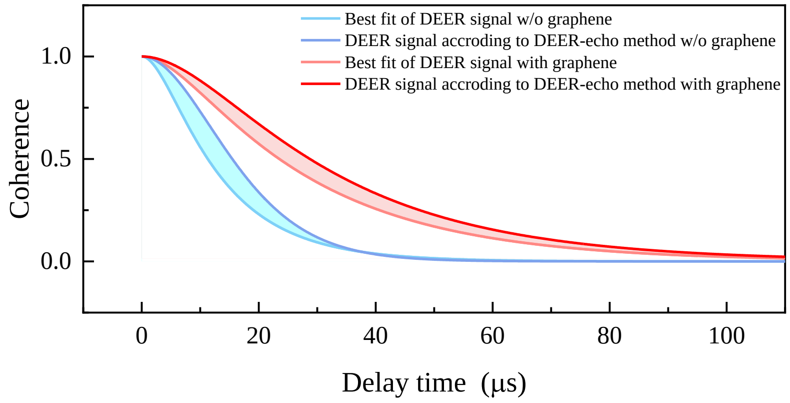

FIG. S4. **The effect of the noise irrelevant to the spin bath.** The light red and light blue lines are fits of the DEER signal normalized with the noise floor of noise spectrum shown in Fig. 2c. The dark red and dark blue lines are the theoretical DEER signal according to Eq. S6 with B_{rms} and τ_c obtained from the DEER-echo method shown in Fig. 3d in the main text. The difference (colored area) is reasonable because it is very challenging to eliminate the decoherence irrelevant to the spin bath completely. However, the small difference results in the totally wrong B_{rms} and τ_c.

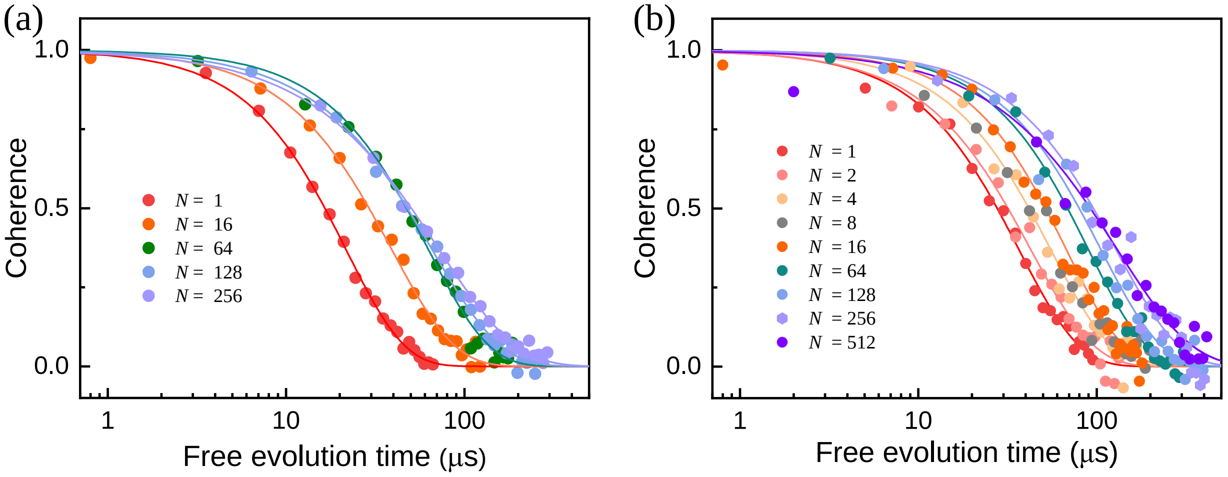

FIG. S5. **The decoherence curves NV1 with and without graphene deposited.** (a) Coherence versus time for different numbers of π pulses without graphene. (b) Coherence versus time for different numbers of π pulses with graphene deposited.

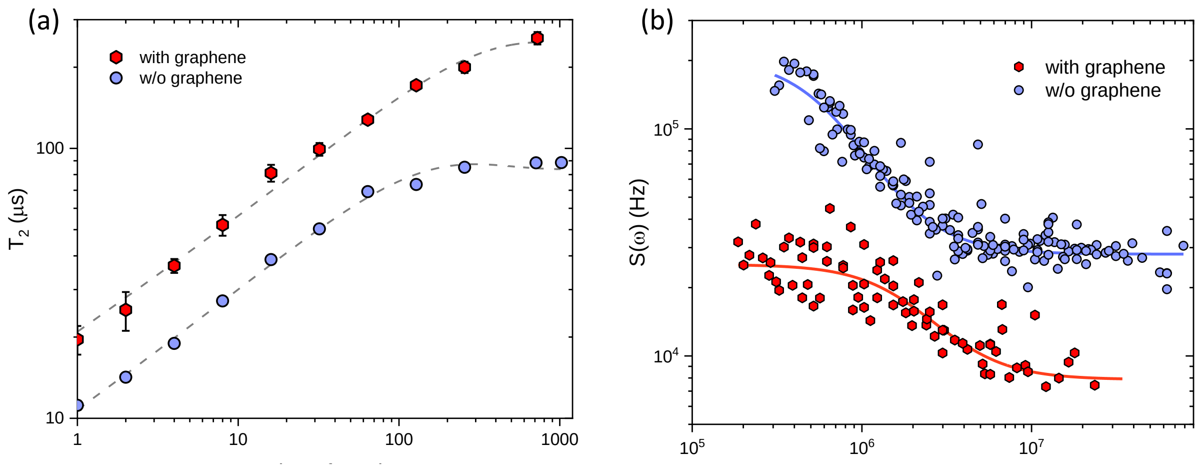

FIG. S6. **Coherence time and noise spectra of a NV with 13.2(6) nm depth (a)** Coherence versus time for different numbers of π pulses with and without graphene. **(b)** Noise spectra extracted from the decoherenc curves using spectral decomposition.

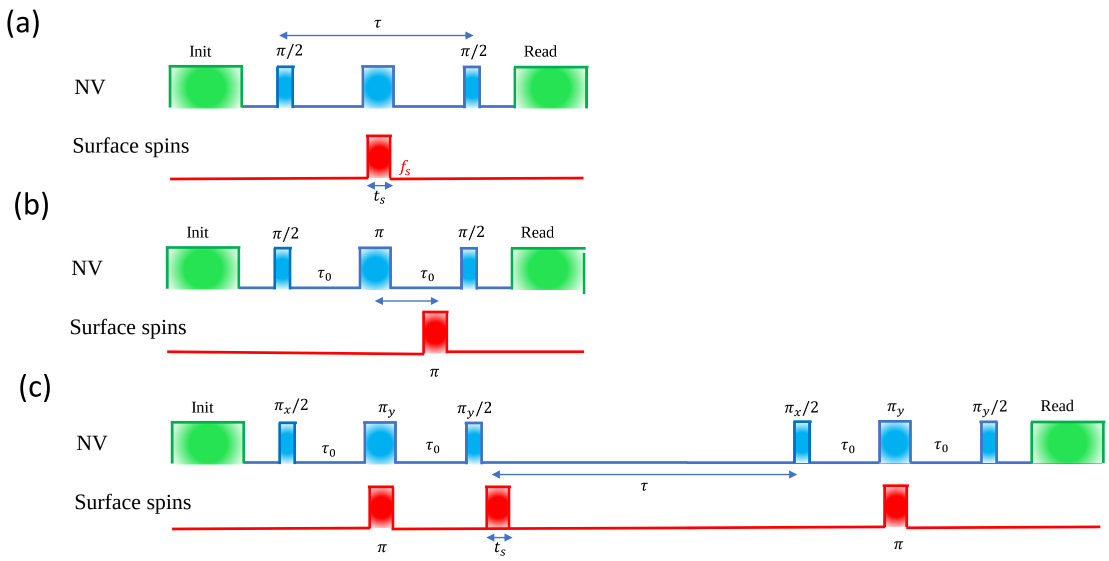

FIG. S7. **Schematic of the pulse sequence.** **(a)** The DEER sequence. **(b)** The DEER-echo sequence. **(c)** The correlation spectroscopy sequence.

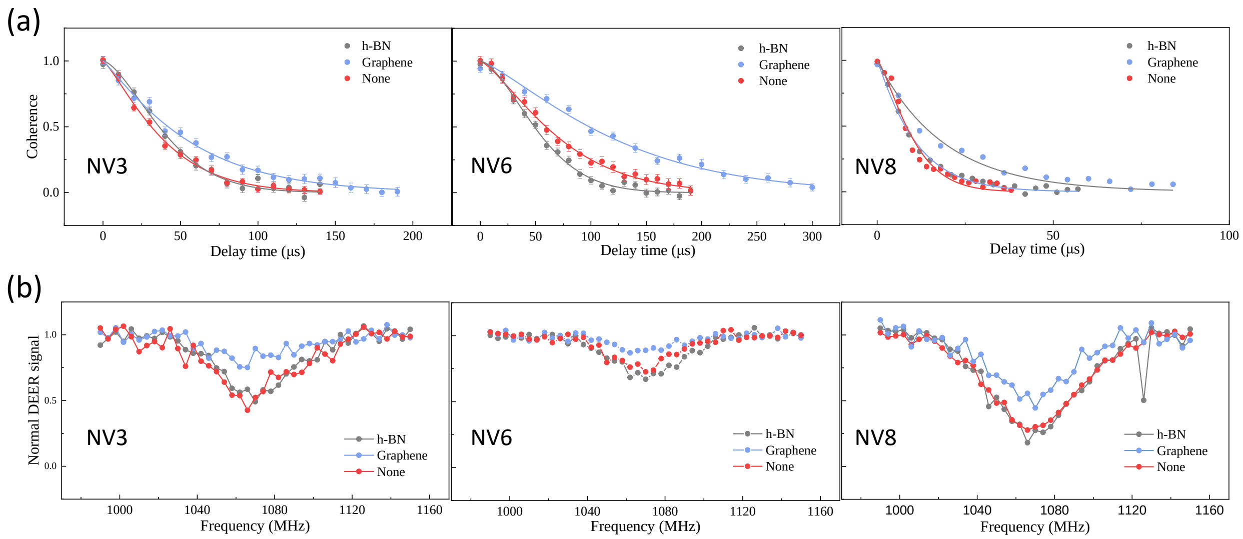

FIG. S8. **Hahn-echo and DEER experiments with h-BN, graphene and nothing deposited.** **(a)**The Hahn-echo decoherence curves. **(b)**The DEER spectra.

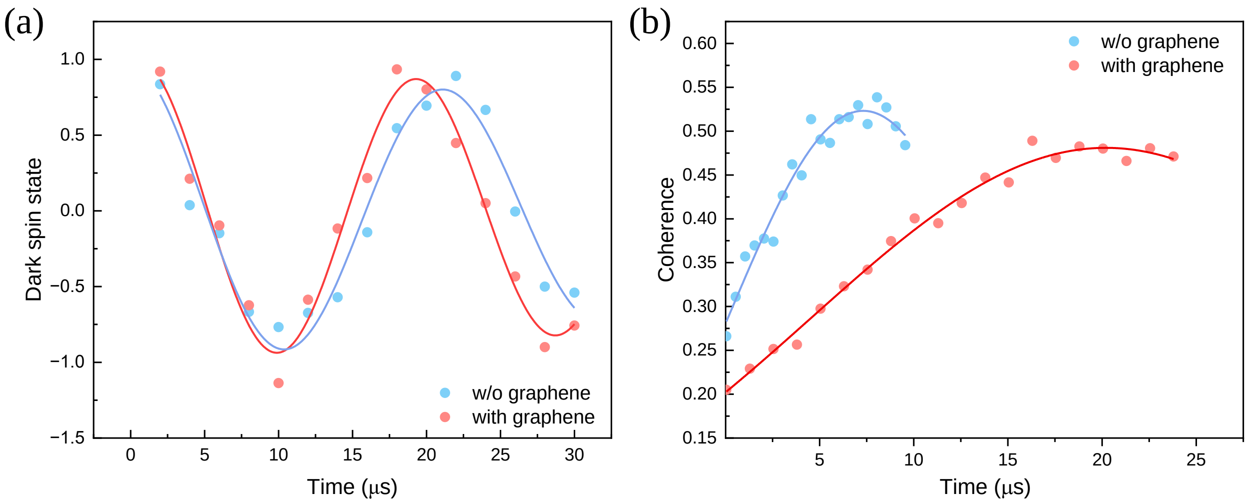

FIG. S9. **The coherence control and DEER-echo measurement of NV with depth of 15.1(4) nm. (A)** Dark spin state as a function of the pulse length t_s. The pulse frequency f_s is 1074 MHz. The solid lines are fits to SineDamp. **(B)** DEER-echo measurement showed a dramatic change in spin density from $1.7(3) \times 10^{-3}$ nm^{-2} to $2.7(2) \times 10^{-4}$ nm^{-2}, corresponding to a notable coherence enhancement of 2.7-folds.

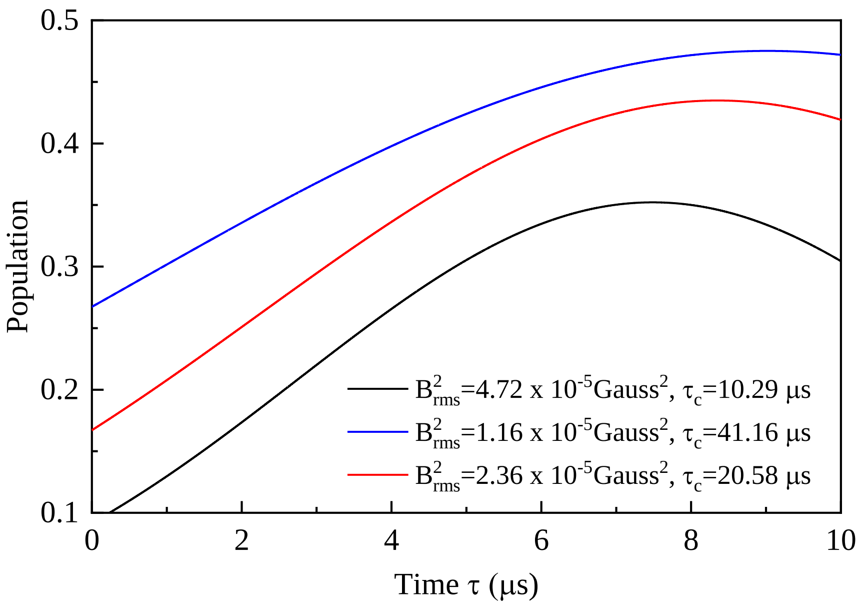

FIG. S10. **Calculation results of DEER-echo curves with different fluctuation magnetic field and correlation time of surfical electron spins.** The coupling strength $\gamma_e^2 B^2_{\rm rms}\tau_c$ is fixed, and τ_c is set to 10.29 μs (black), 41.16 μs (blue), 20.58 μs (red) respectively.

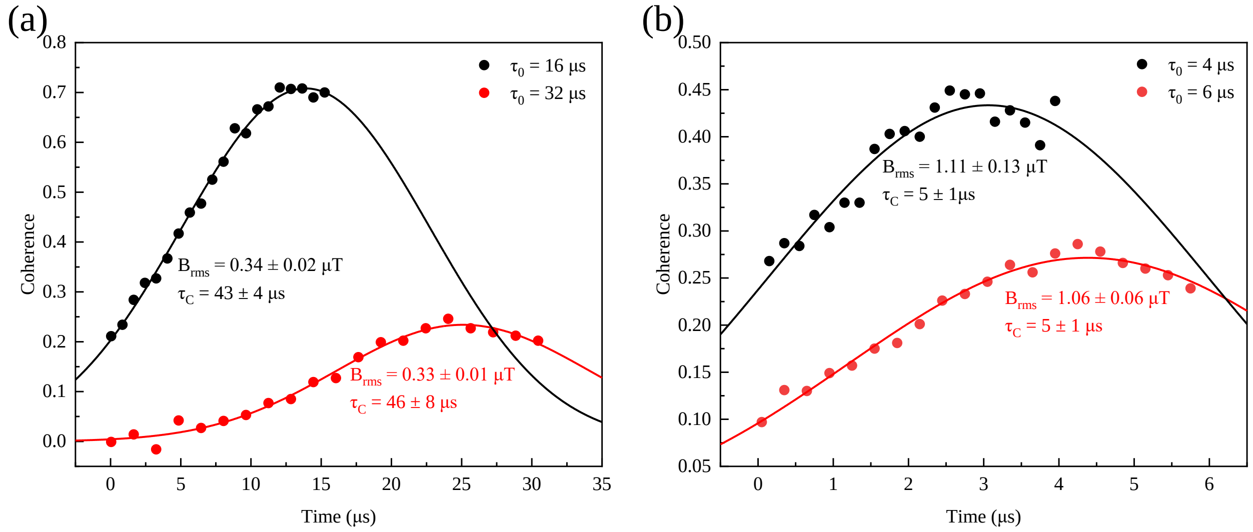

FIG. S11. **DEER-echo experiments with different τ_0. (a)** The experiment results with $\tau_0 = 16\mu s$ (black) and $32\mu s$ (red). **(b)** The experiment results with $\tau_0 = 4\mu s$ (black) and $6\mu s$ (red). The solid lines are fits to the theoretical model according to Eq. S16 and the results are consisted with each other.

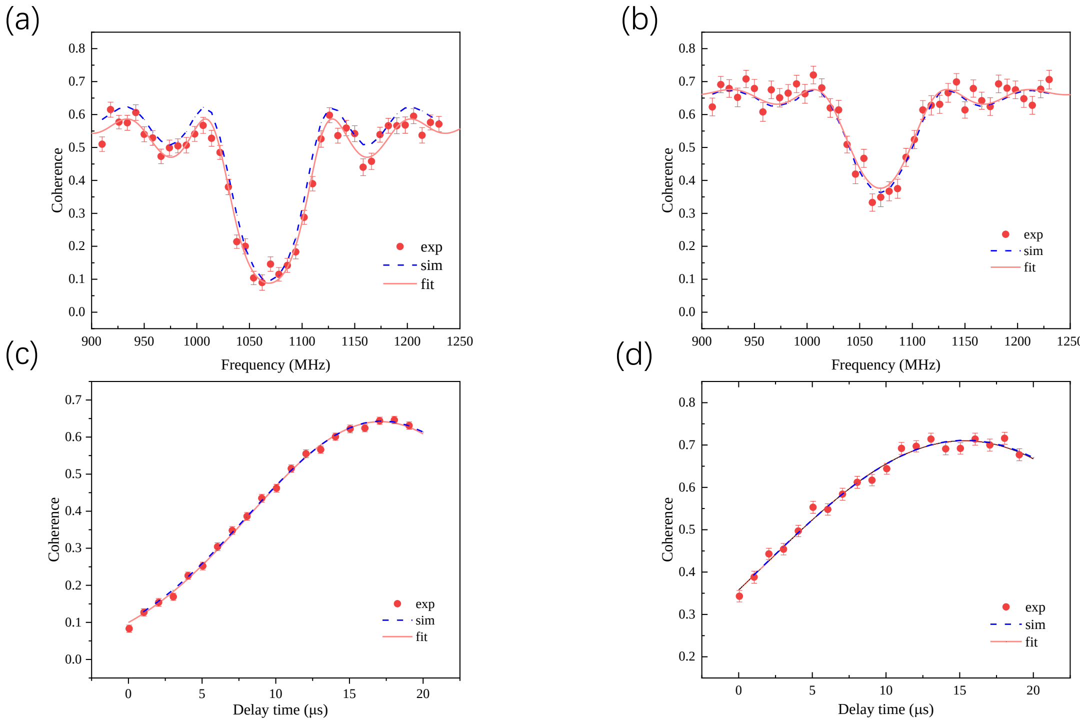

FIG. S12. **Example of the verification of the theoretical model: Deer spectrum and DEER-echo experiment. The depth of the NV is** $21.9 \pm 0.6nm$**.** **(a)** DEER spectrum of NV4 without graphene coating. The NV coherence versus pulse frequency shows a resonance corresponding to g=2 electron spins without graphene coating. The solid line is fitted by the theoretical model (Eq. S14) with the fixed correlation time $\tau_c = 49\ \mu s$. The dashed line is the simulation with the spin density $\sigma = 0.0159$ nm^{-2} and correlation time $\tau_c = 49\ \mu s$. **(b)** DEER spectrum of NV4 with graphene. The NV coherence versus frequency with graphene coating and the dip is reduced. The solid line is fitted by the theoretical model (Eq. S14) with the fixed correlation time $\tau_c = 49\ \mu s$. The dashed line is the simulation with spin density $\sigma = 0.0082$ nm^{-2}and correlation time $\tau_c = 23\ \mu s$. **(c)** The experimental result of the DEER echo sequence performed without graphene. The solid line is a fit to the theoretical model (Eq. S16) with $B_{\rm rms} = 1.08 \pm 0.05\ \mu T$ and $\tau_c = 49 \pm 5\ \mu s$. **(d)** The experimental result of the DEER echo sequence performed with graphene deposited. The solid line is a fit to the theoretical model (Eq. S16) with $B_{\rm rms} = 0.55 \pm 0.06$ μT and $\tau_c = 23 \pm 3\ \mu s$.

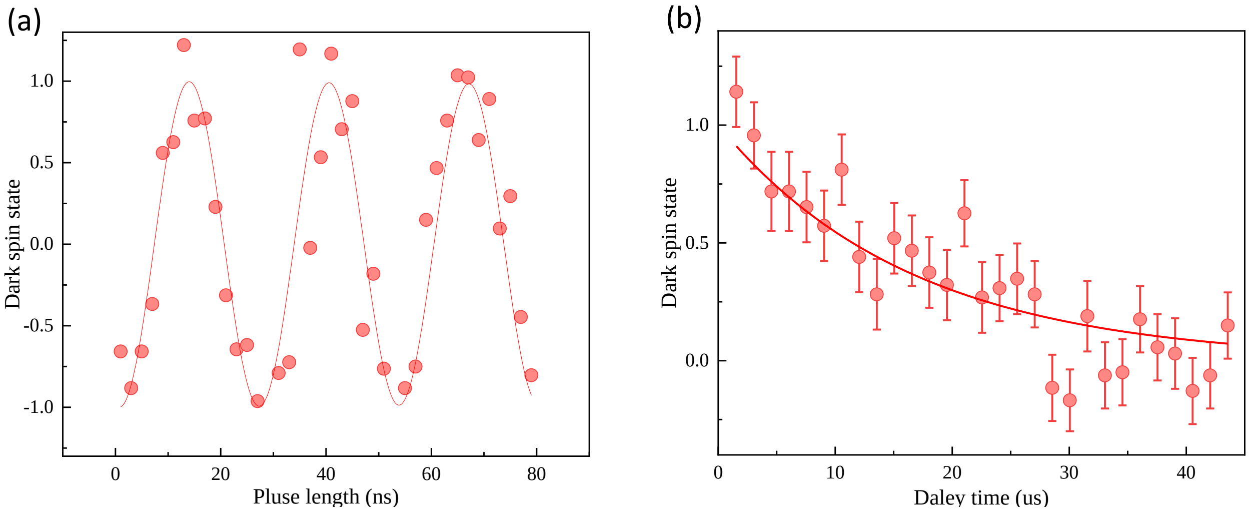

FIG. S13. **The correlation spectrum of NV1 without graphene covering.** **(a)** Spin state of the surface spins as a function of the pulse length t_s. The solid line is a fit to exponentially damped oscillations. **(b)** Spin state of the surface spins as a function of the delay time τ. The solid line is a fit with an exponential decay fit. The fitted τ_c is $17 \pm 2\ \mu$s, which matches the result of the DEER-echo method ($21 \pm 3\ \mu$s).

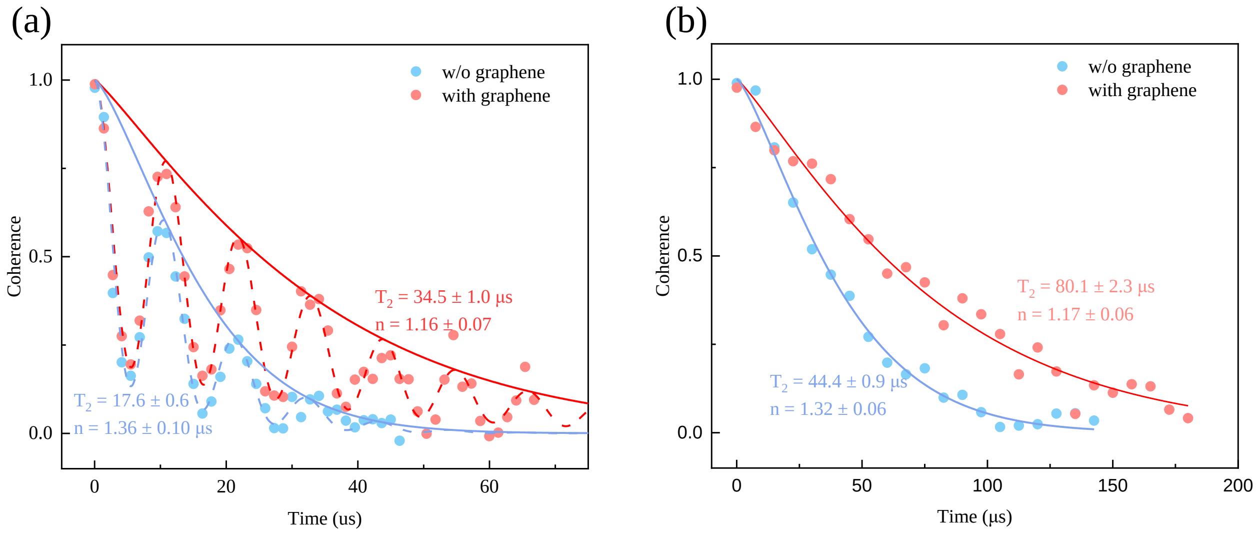

FIG. S14. **Hahn-echo experiments of two different NV centers under other magnetic fields.** **(a)** The Hahn-echo decoherence curves of NV under 172 Gauss. The solid lines are fits of $exp[-(t/T2)^n]$ to the overall envelope of the signal. The dash lines are fits to the observed echo-signal modulations, which are collapses and revivals induced by precession of the natural abundance ^{13}C nuclear-spin bath. **(b)** The Hahn-echo decoherence curves of NV with (red) and without graphene (blue) under 157 Gauss. The signal are fitted with $exp[-(t/T2)^n]$. The two NVs are in two diamond bulk with natural abundance ^{13}C(left) and 0.01% abundance ^{13}C(right) respectively.

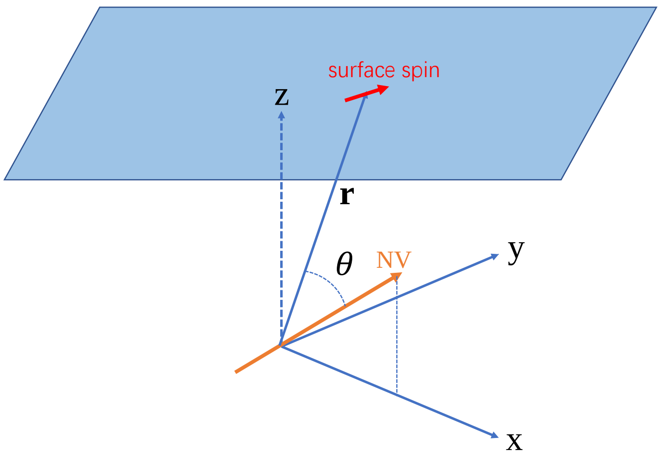

FIG. S15. **Geometry of an NV spin at the origin with its orientation along the [$\sqrt{2}$ 0 1] axis.** The z-axis is defined by the normal of the diamond plane.

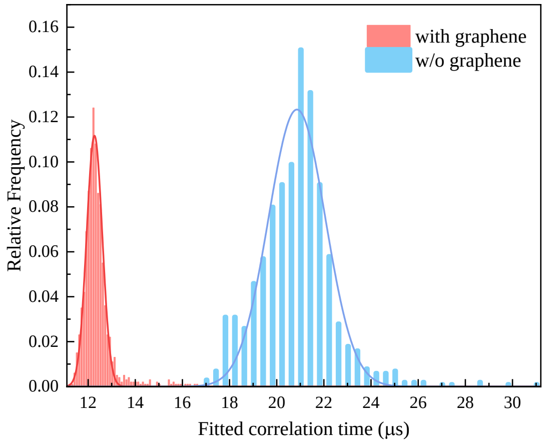

FIG. S16. **The correlation time distribution with dilute surface spins by simulation.** We acquired 1000 simulated results of the DEER-echo sequence under different spin configurations with the same σ and τ_c of NV1 in the main text, and then fitted the simulation curves with the theoretical model shown in Section B.4. The statistical relative frequency of the correlation time τ_c shows two unambiguous distributions corresponding to with (red) and without (blue) graphene. The solid lines are fits to the Gaussian distribution and the standard deviations are 12 ± 1 μs and 21 ± 2 μs.

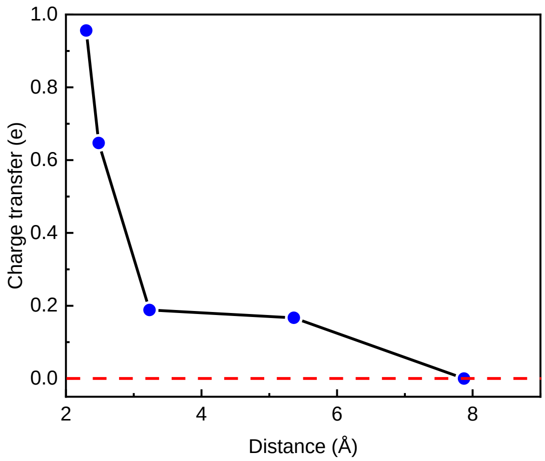

FIG. S17. **The transferred electrons of the graphene-diamond interface as a function of distance.** The charge transfer of the graphene-diamond interface with different initial distances (3 Å, 4 Å, 6 Å, 8 Å and 10 Å) by using Bader charge analysis shows that the transferred electrons of the graphene-diamond interface monotonically decrease with increasing distance.

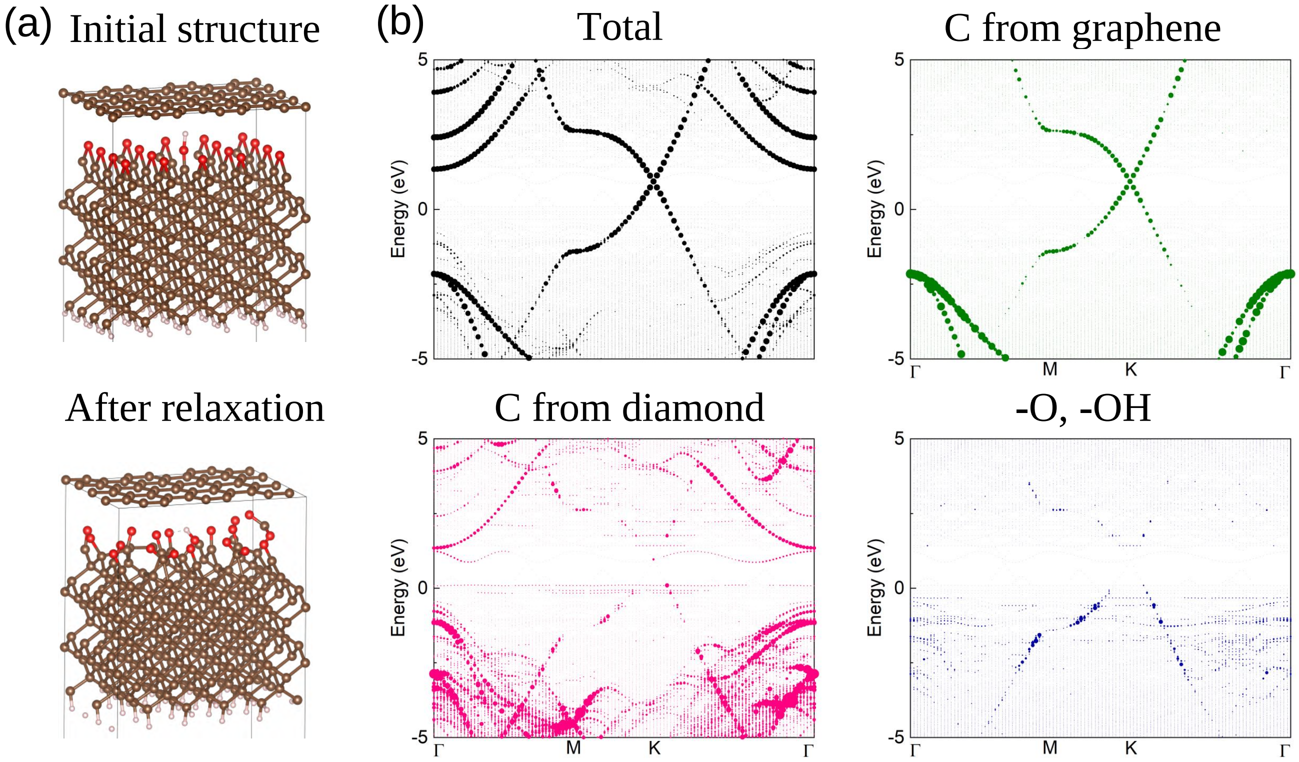

FIG. S18. **The projected band structure of graphene and diamond with O-terminal.** **(a)** The initial structure (upper) and final structure (lower) after relaxation for graphene and diamond with O-terminal. **(b)** The final projected band structure for total, C from graphene, C from diamond and O- or OH-terminal, indicates that diamond has a negligible effect on the energy band structure of graphene. For the structures of diamond with these defects, the band structure that only comprises p orbitals of diamond exhibits typical features of graphene gapped Dirac cones.
